# Supplementary material for: A Denture Use Model Associated with Candida spp. in Immunocompetent Male and Female Rats
Source: J Fungi (Basel). 2022 Apr 30;8(5):466. doi: 10.3390/jof8050466 (PMC9147495; doi:10.3390/jof8050466)
Supplement: Supplementary file 1 [file jof-08-00466-s001.zip › jof-1652815-supplementary.pdf]

Journal of *Fungi*

MDPI

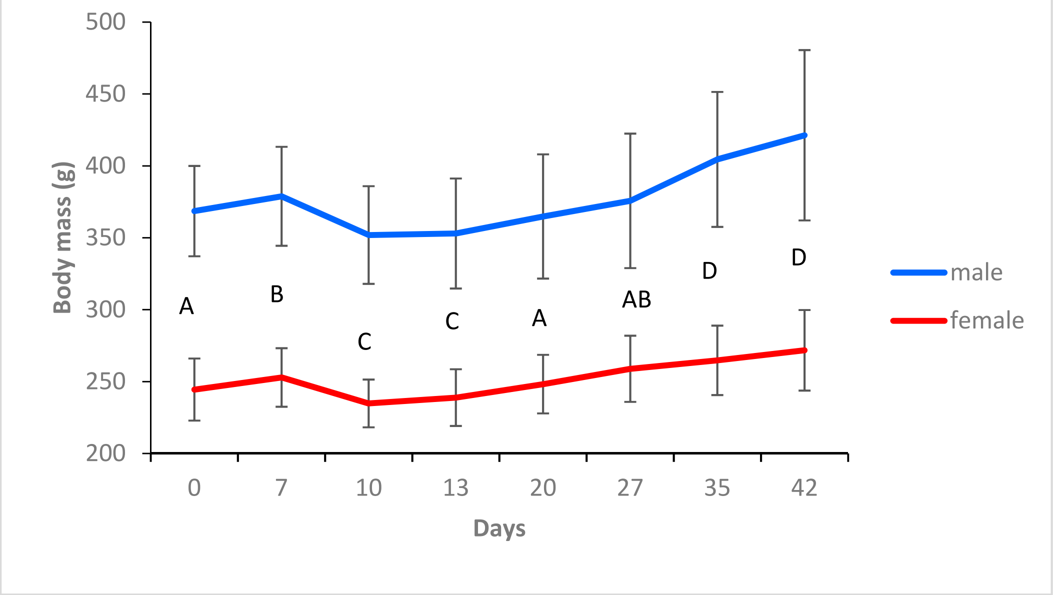

Figure S1. Mean of rats' body mass during the whole experimental period (before and after the cementation of the intraoral acrylic device). Day 0: oral impression; day 7: cementation and first inoculation; day 10: second inoculation; day 13: third inoculation; days 21, 28, and 35: 1st, 2nd and 3rd week after inoculations, respectively; and day 42: euthanasia. Significant difference ($p < 0.001$) was found between male and female rats; different letters denote significant difference ($p \leq 0.006$) among the periods (repeated measures ANOVA). Error bars: standard deviation.

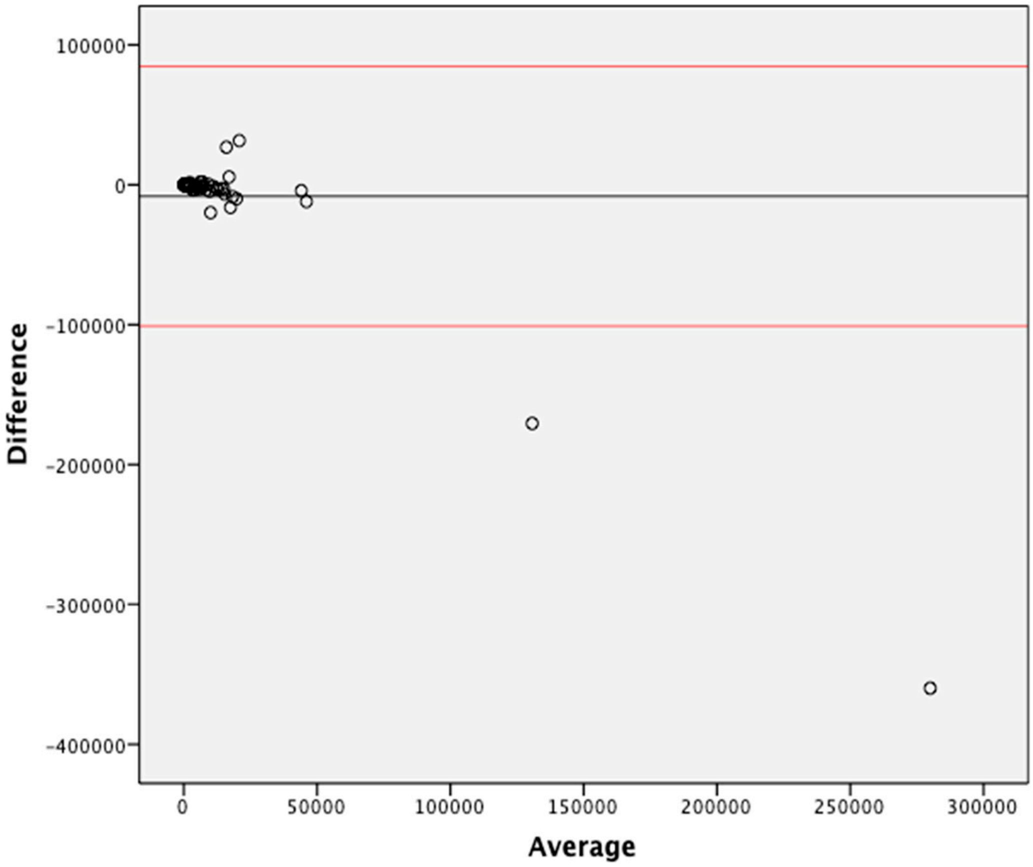

(**a**)

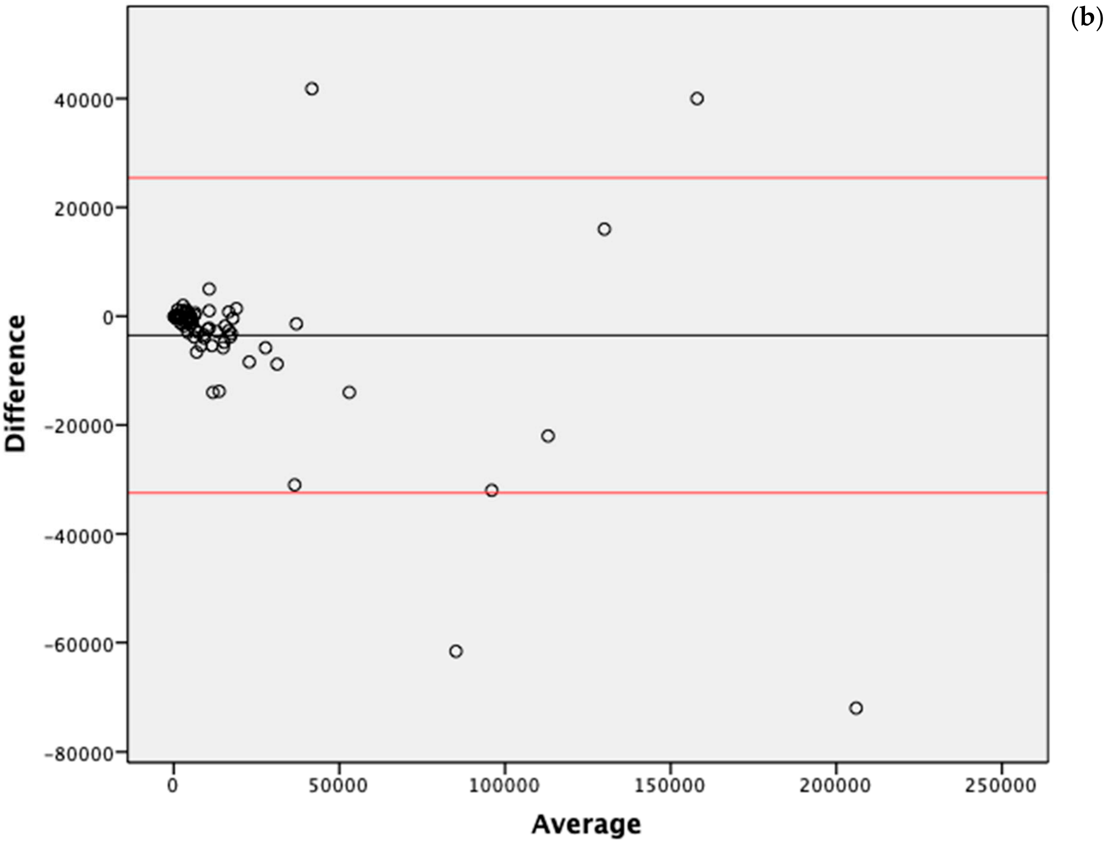

Figure S2. Bland-Altman plots of difference against average (agreement analysis) between CFU/mL values from SDA and CHROMAgar *Candida* recovered from palate (**a**) and device (**b**). The black line shows the mean of difference and the red lines show the upper and lower limits.

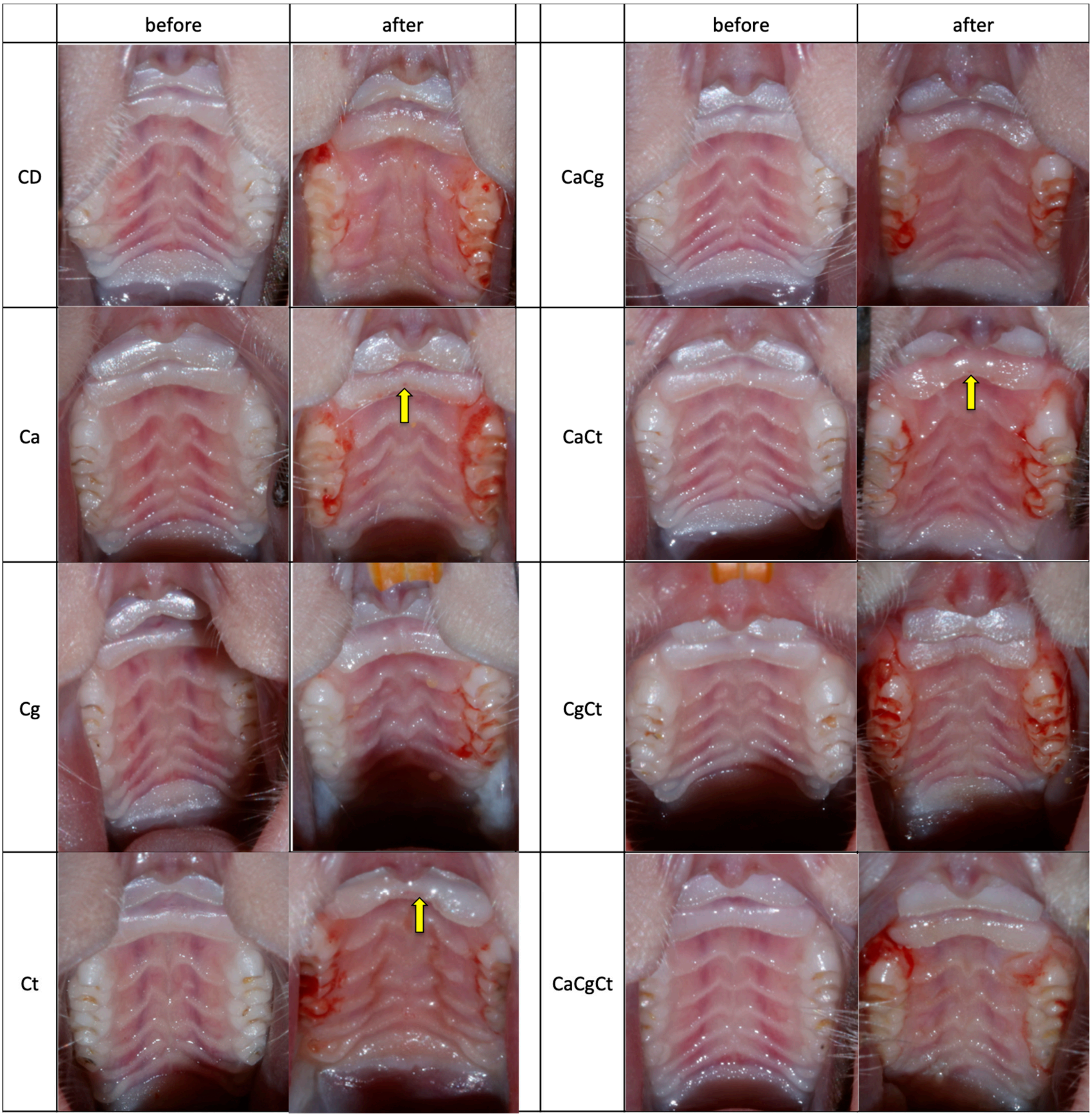

Figure S3. Photographs of the palate of male rats before (day 0) and after (day 42) the 4-week period with the acrylic device. In each group, both photographs (before and after) are from the same animal. The yellow arrows show red spots on the anterior papillae. CD: device control, Ca: *Candida albicans*, Cg: *Candida glabrata*, Ct: *Candida tropicalis*, CaCg: *C. albicans* + *C. glabrata*, CaCt: *C. albicans* + *C. tropicalis*, CgCt: *C. glabrata* + *C. tropicalis*, and CaCgCt: *C. albicans* + *C. glabrata* + *C.tropicalis* groups.

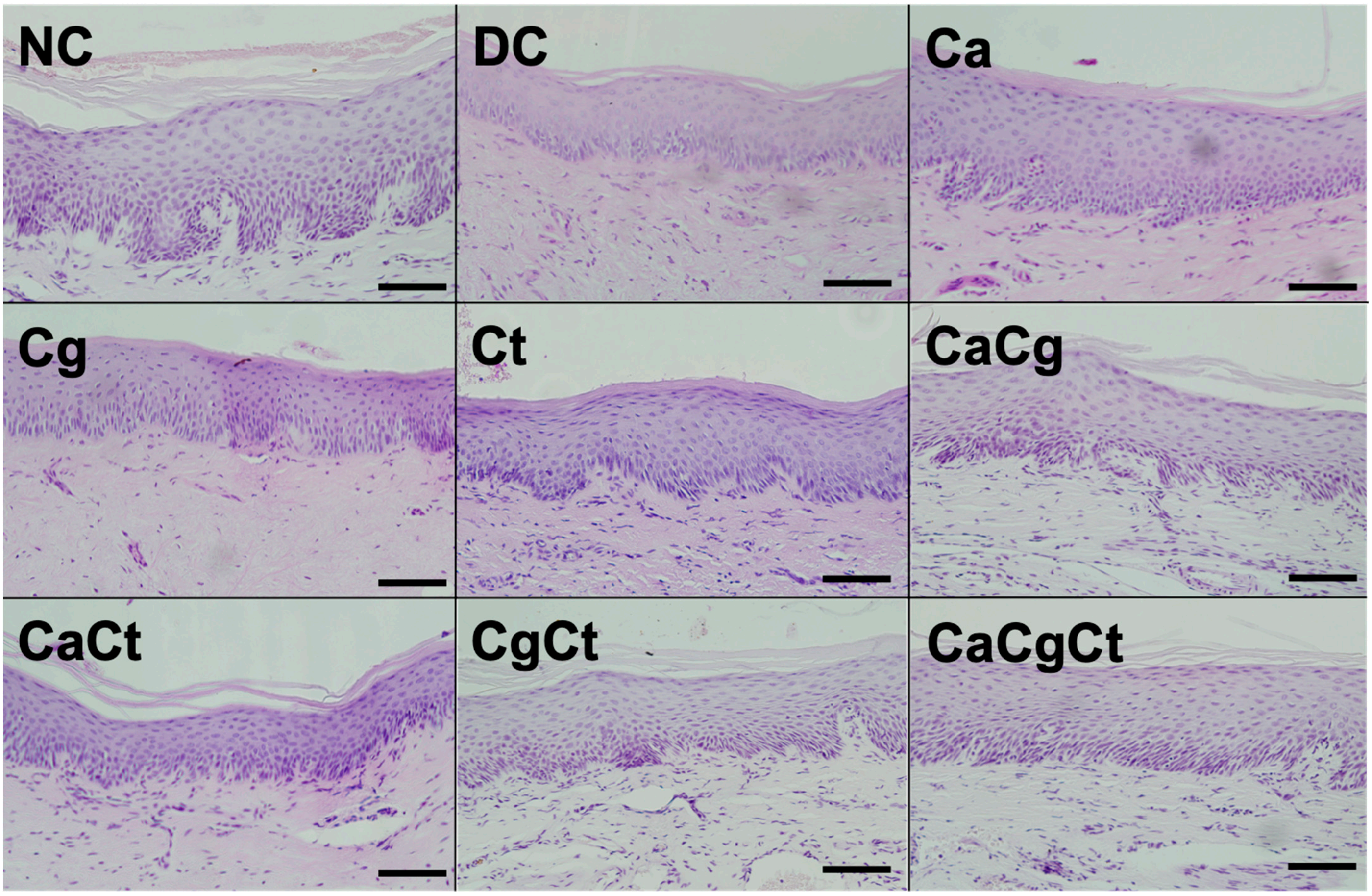

Figure S4. Histopathological sections of the palatal mucosa of male rats after the 4-week period with the acrylic device. NC: negative control; DC: device control, Ca: *C. albicans*, Cg: *C. glabrata*, Ct: *C. tropicalis*, CaCg: *C. albicans* + *C. glabrata*, CgCt: *C. glabrata* + *C. tropicalis*, CaCt: *C. albicans* + *C. tropicalis*, and CaCgCt: *C. albicans* + *C. glabrata* + *C. tropicalis*. Magnification: 20x. Scale bar: 100 µm.
